# Taxonomic Revisiting and Phylogenetic Placement of Two Endangered Plant Species: *Silene leucophylla* Boiss. and *Silene schimperiana* Boiss. (Caryophyllaceae)

**DOI:** 10.3390/plants10040740

**Published:** 2021-04-09

**Authors:** Ahmed EL-Banhawy, Iman H. Nour, Carmen Acedo, Ahmed ElKordy, Ahmed Faried, Widad AL-Juhani, Ahmed M. H. Gawhari, Asmaa O. Olwey, Faten Y. Ellmouni

**Affiliations:** 1 Botany and Microbiology Department, Faculty of Science, Suez Canal University, 41522 Ismailia, Egypt; 2 Botany and Microbiology Department, Faculty of Science, Alexandria University, 21511 Alexandria, Egypt; 3 Biodiversity and Environment Management Department, Faculty of Biological and Environmental Sciences, University of León, 24071 León, Spain; c.acedo@unileon.es (C.A.); aelkordy@science.sohag.edu.eg (A.E.); 4 Botany and Microbiology Department, Faculty of Science, Sohag University, 82524 Sohag, Egypt; 5 Botany and Microbiology Department, Faculty of Science, Assiut University, 71515 Assiut, Egypt; ahmedfaried55@aun.edu.eg (A.F.); asmaa_olwey@science.aun.edu.eg (A.O.O.); 6 Biology Department, College of Science and Arts, Sajir, Shaqra University, 11961 Shaqra, Saudi Arabia; 7 Biology Department, Faculty of Applied Science, Umm Al-Qura University, 24381 Makkah, Saudi Arabia; wsjuhani@uqu.edu.sa; 8 Research Laboratories Centre, Faculty of Applied Science, Umm Al-Qura University, 24381 Makkah, Saudi Arabia; 9 Botany Department, Faculty of Science, University of Benghazi, 1308 Benghazi, Libya; ahmed.gawhari@gmail.com; 10 Botany Department, Faculty of Science, Fayoum University, 63514 Fayoum, Egypt; fyl00@fayoum.edu.eg

**Keywords:** endangered, endemic, *Silene*, SEM, stomata, molecular systematics, phylogenetic analysis, nrDNA ITS, cpDNA *matk*, *Siphonomorpha*, *Sclerocalycinae*

## Abstract

The genus *Silene* L. is one of the largest genera in Caryophyllaceae, and is distributed in the Northern Hemisphere and South America. The endemic species *Silene leucophylla* and the near-endemic *S. schimperiana* are native to the Sinai Peninsula, Egypt. They have reduced population size and are endangered on national and international scales. These two species have typically been disregarded in most studies of the genus *Silene*. This research integrates the Scanning Electron Microscope (SEM), species micromorphology, and the phylogenetic analysis of four DNA markers: ITS, *mat*K, *rbc*L and *psb*-A/*trn-*H. Trichomes were observed on the stem of *Silene leucophylla,* while the *S. schimperiana* has a glabrous stem. Irregular epicuticle platelets with sinuate margin were found in *S. schimperiana*. Oblong, bone-shaped, and irregularly arranged epidermal cells were present on the leaf of *S. leucophylla,* while *Silene schimperiana* leaf has “tetra-, penta-, hexa-, and polygonal” epidermal cells. *Silene leucophylla* and *S. schimperiana* have amphistomatic stomata. The Bayesian phylogenetic analysis of each marker individually or in combination represented the first phylogenetic study to reveal the generic and sectional classification of *S. leucophylla* and *S. schimperiana*. Two *Silene* complexes are proposed based on morphological and phylogenetic data. The *Leucophylla* complex was allied to section *Siphonomorpha* and the *Schimperiana* complex was related to section *Sclerocalycinae*. However, these two complexes need further investigation and more exhaustive sampling to infer their complex phylogenetic relationships.

## 1. Introduction

Caryophyllaceae contain 70–86 genera and 2200 species, which are distributed all over the world [1]. The family is divided into four subfamilies: Alsinoideae, Caryophylloideae, Paronychioideae, and Polycarpoideae “Polycarpaoideae” [2]. Within the Caryophylloideae, the tribe Sileneae DC. is regarded as the largest tribe in the family [3].

*Silene* L. is one of the largest genera in Caryophyllaceae, with about 850 species, distributed throughout Eurasia, from temperate regions of the Mediterranean basin to central and western Asia [4,5]. The genus *Silene* L. is divided into three subgenera, *Lychnis* (L.), *Behenantha* (Otth) Torr. & A. Gray, and *Silene* (Rohrbach) [6], as well as 34 sections, based on morphological and phylogenetic analyses [4].

Egypt’s Sinai Peninsula is a central area between Africa and Asia with a unique ecosystem [7,8]. South Sinai is home to 14 endemic and threatened plant species [5]. Twenty-nine *Silene* taxa are native and well recognized in Egypt; two are endemic, *Silene leucophylla* Boiss. and *S. oreosinaica* Chowdhuri, while *Silene schimperiana* Boiss. is near-endemic [9,10,11].

*Silene leucophylla* and *S. schimperiana* are two perennial hemicryptophytes. They are isolated at high elevations (1775–2099 m) and grow in the Saint Katherine Protectorate Mountains’ rocky habitats in Southern Sinai.

*Silene leucophylla* Boiss. and *S. schimperiana* are accepted names. These names were derived from World Checklist of Selected Plant Families (WCSP). No synonyms are recorded for either taxa [12]. *Silene leucophylla* is a critically endangered species with a reduced population size, i.e., 109 individuals [13], whereas, *S. schimperiana* has been evaluated as endangered at a national scale [14]. The relatively small populations are susceptible to threats such as overgrazing and environmental changes [15]. The chromosome count and karyotype study was performed for *S. schimperiana*, it was (2n = 2x = 24) [16]. Therefore, the wild population of *Silene* species could be under severe ecological pressures that would lead to their extinction [14].

Rohrbach [6] classified *S. leucophylla* and *S. schimperiana* in the same section, “*Botryosilene*”. While he considered *S. leucophylla* in the Nutantes series, *S. schimperiana* was classified by him into series *Sclerocalycinae*. Classification of the genus *Silene* by [10,11] considered *S. leucophylla* a member of section *Siphonomorpha*, while *S. schimperiana* was allied to section *Sclerocalycinae* (Subsection *Chlorifoliae*).

The infraspecific classification of the genus *Silene* based on morphological description showed that *Silene leucophylla* and *S. schimperiana* allied to subgenus *Silene* sections Siphonomorpha Otth and section Sclerocalycinae Boiss., respectively [9,10,11]. A table of morphological differences between *Silene leucophylla* and *Silene schimperiana* and the nearest elements of the genus *Silene* is represented in the Supplementary (Table S1).

Scanning Electron Microscopy (SEM) has a vital role in the discrimination between taxa within the genus [17,18]. Moreover, the stomatal distribution pattern is highly variable among *Silene* species and represents another powerful tool for species discrimination [19,20,21,22,23,24,25].

Phylogenetic analysis is essential for explaining structural, ecological, taxonomical, and functional biodiversity characteristics in an evolutionary background [26,27]. The critical species *Silene leucophylla* and *S. schimperiana* have been disregarded in recent phylogenetic and taxonomic studies of the genus *Silene* [4,28].

The present study explores the micromorphological characterization and molecular phylogeny of *S. leucophylla* and *S. schimperiana*, revealing the phylogenetic placement of these species and helping to resolve sectional classification within the whole genus.

## 2. Results

### 2.1. Scanning Electron Microscope (SEM)

#### 2.1.1. Stem Micromorphology

Pustulate unicellular non-glandular trichomes of (45–98 × 13–23 µm) were observed on the stem of *Silene leucophylla*. In comparison, *S. schimperiana* had a glabrous stem surface. A thin layer of epicuticular wax was found in *S. leucophylla*. Simultaneously, *S. schimperiana* was covered with irregular crustose platelets of <1 µm height, with sinuate margin.

**Figure 1:**
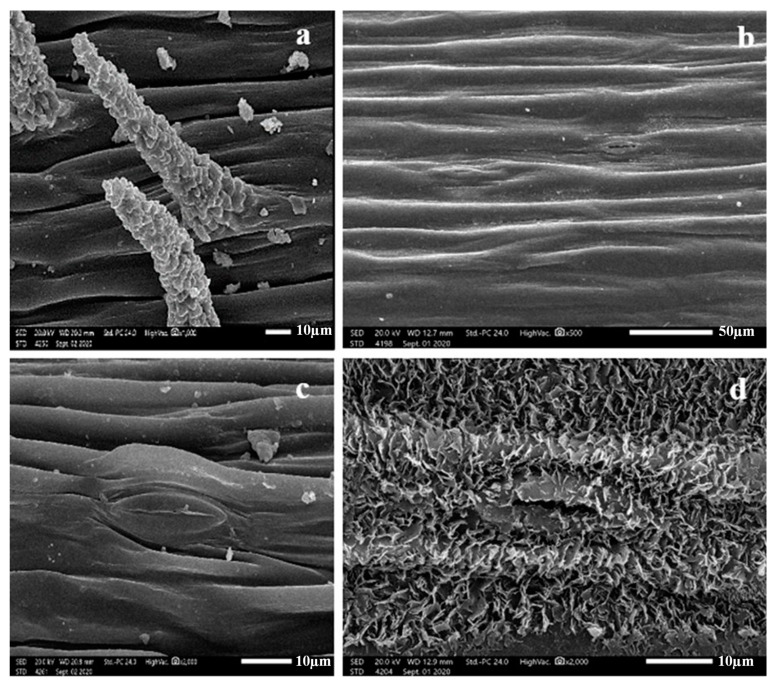
Scanning Electron Microscope (SEM) photomicrographs of *Silene* stem. Stem texture: (**a**) Non-glandular pustulate trichome of *Silene leucophylla*; (**b**) glabrous stem surface of *Silene schimperiana*. Stem epicuticular wax: (**c**) thin layer with anomocytic stomata of *Silene leucophylla*; (**d**) irregular epicuticular crustose platelets with anomocytic stomata in *Silene schimperiana*.

The type of stomatal complex was anomocytic in both species (Figure 1). Stem qualitative and quantitative traits are summarized in Table 1 and Supplementary (Table S2).

**Figure 2:**
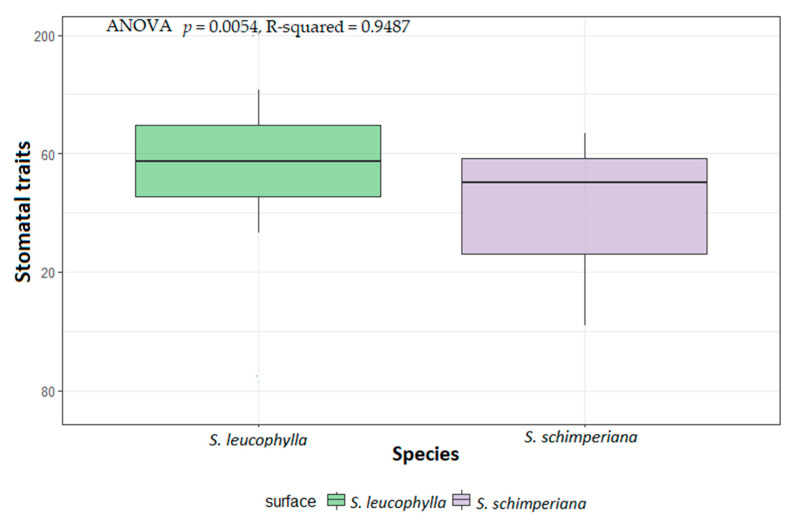
Boxplots of micromorphological stem stomatal traits of *Silene leucophylla* and *Silene schimperiana*.

Geom-boxplot and ANOVA indicate a significant variation of stomatal traits among the two species with *p*-value (*p* = 0.0054, R-squared = 0.9487). Higher median stem stomata of *S. leucophylla* than *S. schimperiana* was also recorded in (Figure 2).

#### 2.1.2. Leaf Epidermal Cells

**Figure 3:**
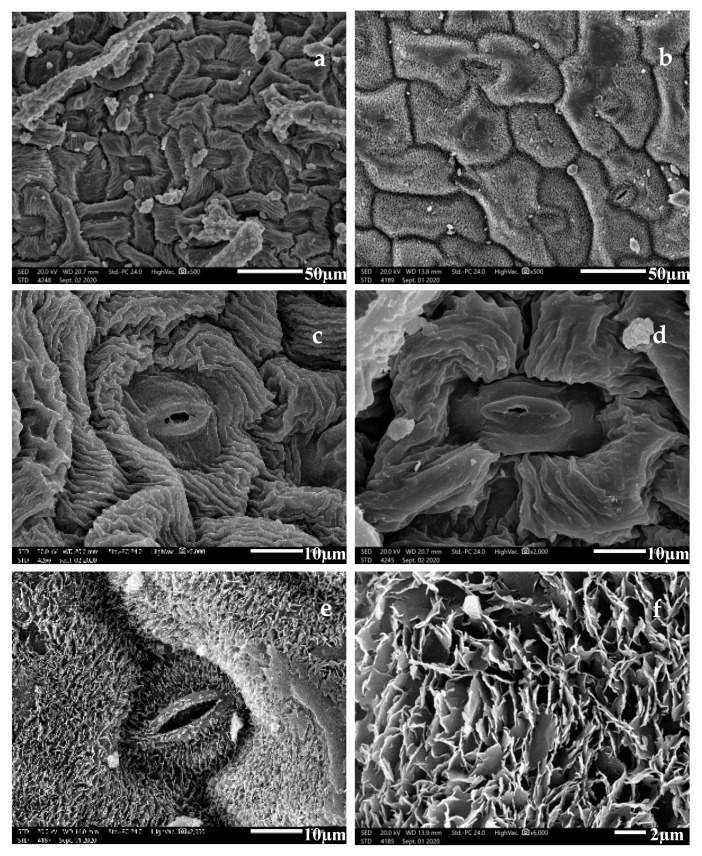
Scanning Electron Microscope (SEM) photomicrographs of *Silene* leaf surfaces. Epidermal cell shapes in (**a**) *Silene leucophylla* and (**b**) *Silene schimperiana*. Ribbing pattern of fine relief in *Silene leucophylla*: (**c**) densely ribbed and raised stomata, (**d**) moderately ribbed. Epicuticular platelets in *Silene schimperiana*: (**e**) with sunken stomata, (**f**) irregular platelets. (Abaxial leaf surface (**b**,**c**,**e**); Adaxial leaf surface (**a**,**d**,**f**).

The leaf epidermal cells of the adaxial (AD) and abaxial (AB) surface of *S. leucophylla* were oblong, bone-shaped, and irregularly arranged. On the other hand, in *S. schimperiana*, the AD and AB surfaces’ epidermal cells were “tetra-, penta-, hexa-, and polygonal” parallelly arranged (Figure 3).

**Figure 4:**
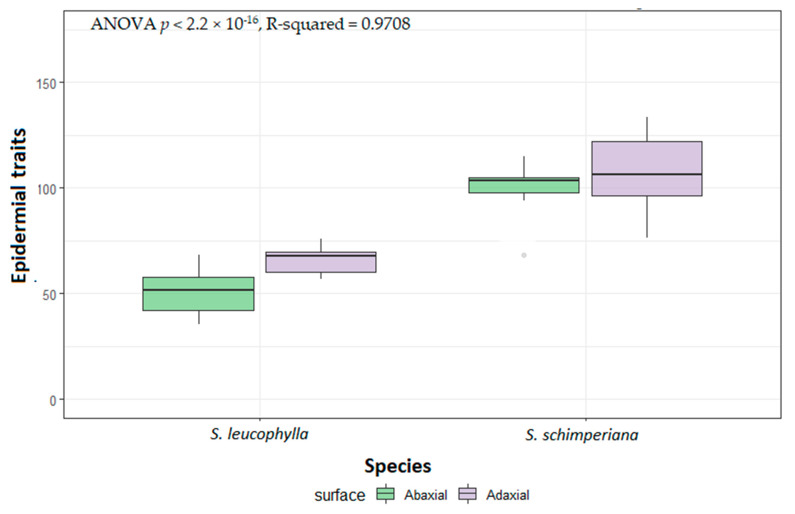
Boxplots of micromorphological quantitative measurements of epidermal cells of *Silene leucophylla* and *Silene schimperiana* leaves.

The anticlinal walls (AW) were sunken, irregularly channeled and curved in *S. leucophylla*. At the same time, it was sunken but straight in *S. schimperiana*. The fine relief of the epidermal cell wall was highly ribbed on the A.B. surface and moderately ribbed on the AD surface in *S. leucophylla*. In contrast, it was covered by irregular epicuticular crustose platelets in *S. schimperiana* (Figure 3). Significant variations in the size of leaf epidermal cells were recorded by ANOVA analysis for the two species (*p* < 2.2 × 10^−16^, R-squared = 0.9708). The smallest stomatal area was recorded on the AB surface of *S. leucophylla* (46.44–74.64 = 61.42 ± 8.56 µm^2^), whereas the largest area was recorded on the AD surface of *S. schimperiana* (102.20–253.50 = 152.30 ± 37.92 µm^2^). Those measurements were confirmed by grouped boxplot for abaxial and adaxial leaf (Figure 4).

#### 2.1.3. Stomatal Complex

Leaves of the studied *Silene* taxa were amphistomatic. They have raised diacytic stomata with smooth guard cells observed in *S. leucophylla*, while *S. schimperiana* attained sunken diacytic stomata with irregular epicuticular crustose platelets on guard cells (Figure 3).

**Figure 5:**
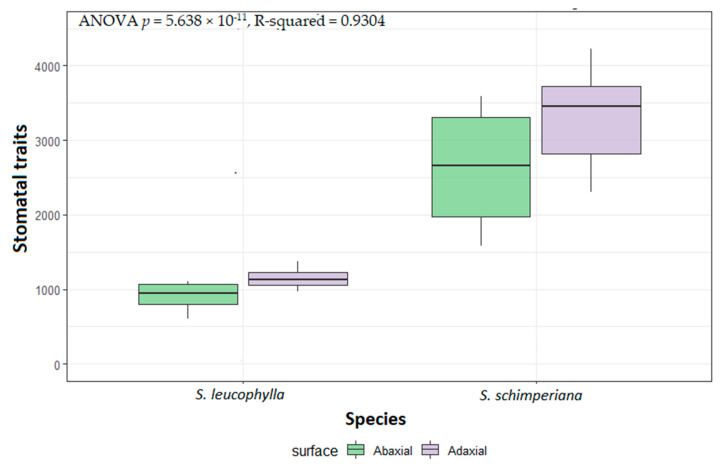
Boxplots of the quantitative data of complex stomatal characteristics, subsidiary cells and stomatal pore measurements of *Silene leucophylla* and *Silene schimperiana*.

The smallest stomatal area was recorded on the AB surface of *S. leucophylla* (46.44–74.64 = 61.42 ± 8.56 µm^2^), On the other hand, the largest stomatal area was measured on the AD surface of *S. schimperiana* (102.20–253.50 = 152.30 ± 37.92 µm^2^) (Figure 5).

ANOVA of the length, width and area for the stomatal pore, stomatal complex and subsidiary cells of both AB and AD were *p* = 5.638 × 10^−11^, R-squared = 0.9304. Grouped boxplot for those measurements were represented in (Figure 5).

**Figure 6:**
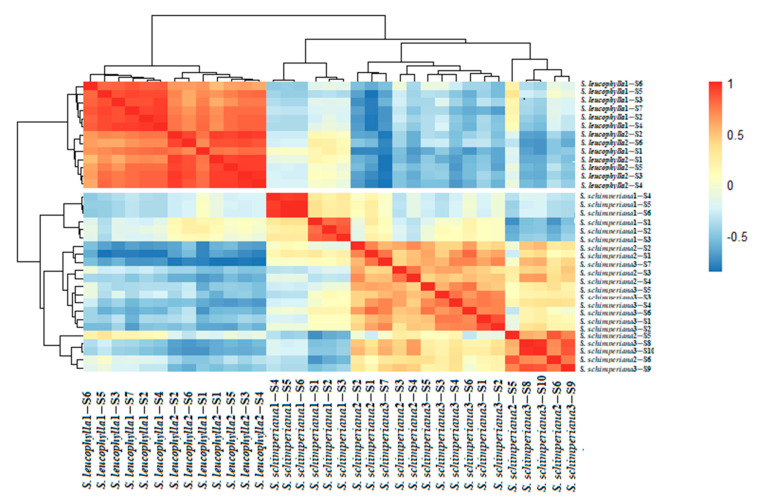
Pheatmap based on the quantitative data of stem and leaf micromorphological traits, showing the similarity and dissimilarity within and among *Silene leucophylla* and *Silene schimperiana*.

**Figure 7:**
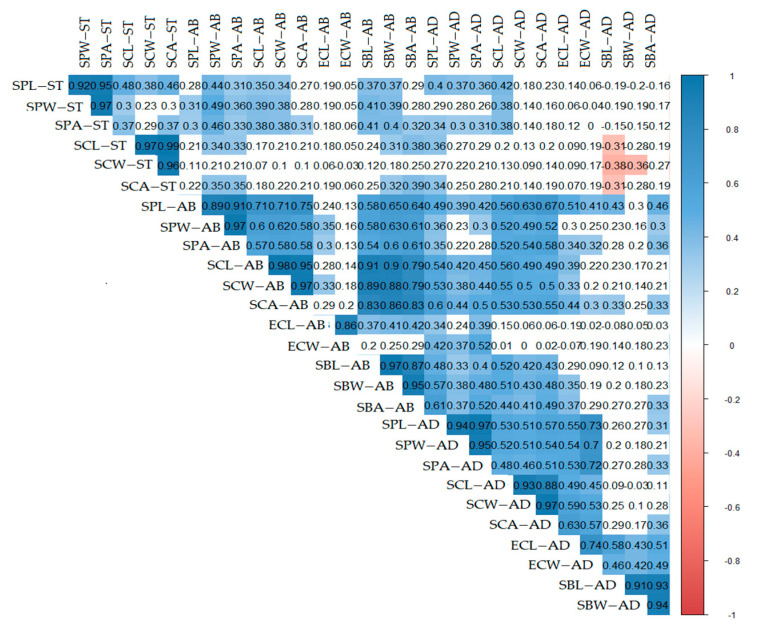
Correlogram between quantitative traits of *Silene leucophylla* and *Silene schimperiana*. Positive and negative correlations are displayed in blue and red color, respectively. Correlation coefficients are proportional to color intensity.

The lowest stomatal index (SI%) was recorded in *S. leucophylla* (11.76–12.12 = 11.94 ± 0.25), whereas the highest SI (12.90–20.69 = 15.10 ± 3.76) was noticed for the AD surface in *S. schimperiana.* The pheatmap in (Figure 6) represents the overall variations between the investigated taxa. *S. leucophylla* representatives grouped in a separate cluster diverting from representatives of *S. schimperiana*. The correlogram correlation analysis showed a significant relationship among numerous traits; (Figure 7).

### 2.2. Phylogeny

The phylogenetic placement of the Egyptian species *S. leucophylla* and *S. schimperiana* within the whole genus was conducted using novel DNA sequences. The used markers were: a nuclear marker “nrDNA” Internal Transcribed Spacer (ITS), and three plastid markers “cpDNA”: *mat*K; *psb*A-*trn*H, and *rbc*L.

The matrices of the DNA sequences of ITS, *mat*K, *psb*-A/*trn*-H, and *rbc*L consisted of 48, 34, 36, and 23 *Silene* taxa, respectively. The outgroups were *Petrocoptis glaucifolia*, *P. viscosa* and *P. pyrenaica* in the ITS tree. *Agrostemma githago* and *Petrocoptis pyrenaica* were used as outgroups in *mat*K. *Bufonia multiceps* was used in both *psb*-A*/trn*-H and and *rbc*L.

**Figure 8:**
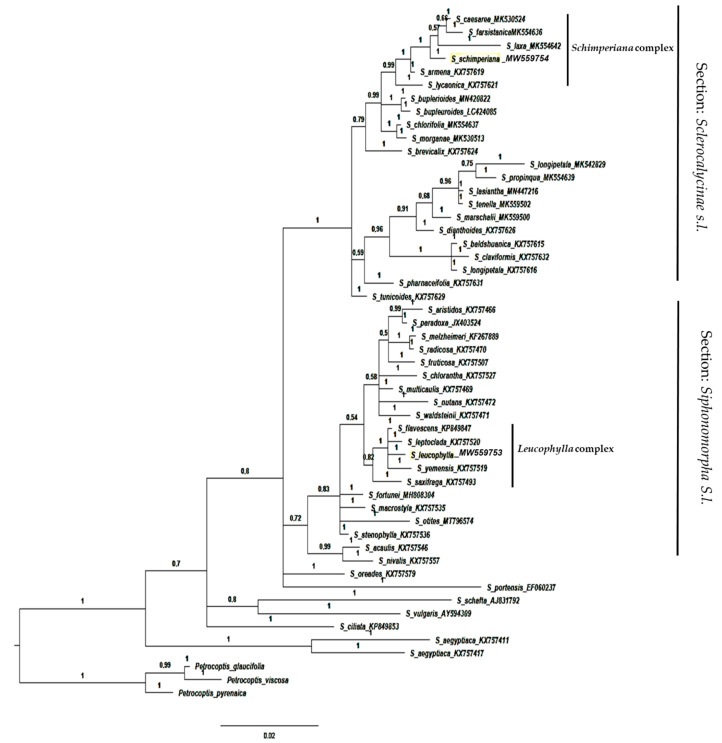
Bayesian phylogenetic tree of *Silene* species based on nrDNA sequences of the ITS marker. Numbers above branches represented (PP).

**Figure 9:**
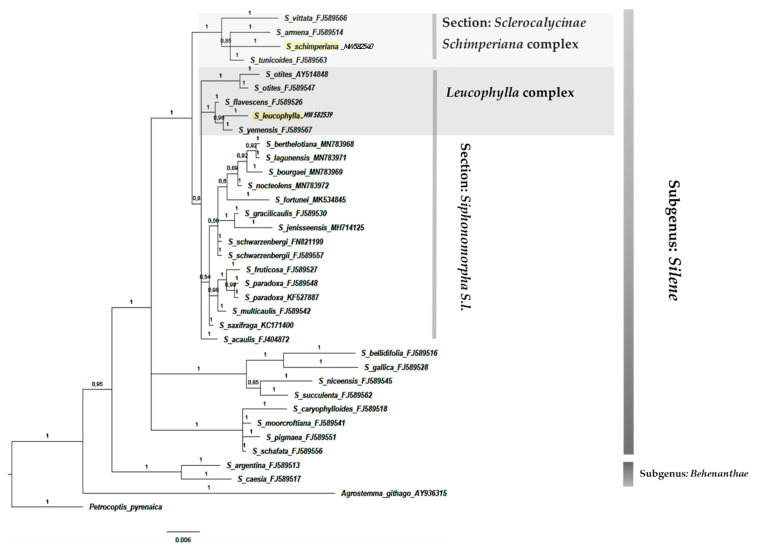
Bayesian phylogenetic tree of *Silene* species based on cpDNA sequences of the *mat*K marker. Numbers above branches represented (PP).

The results showed that section *Sclerocalycinae* represented a well-supported monophyletic clade (PP = 1) composed of 22 *Silene* taxa in the ITS analysis (Figure 8) and four taxa in the *mat*K (Figure 9). The Egyptian species *S. schimperiana* was related to this section.

Section *Siphonomorpha* was also retrieved successfully. It consisted of 20 *Silene* including the Egyptian endemic species *S. leucophylla*. This section was moderately supported in both phylogenies PP = 0.72 in the ITS (Figure 8), and in the *mat*k PP = 0.8 (Figure 9).

**Figure 10:**
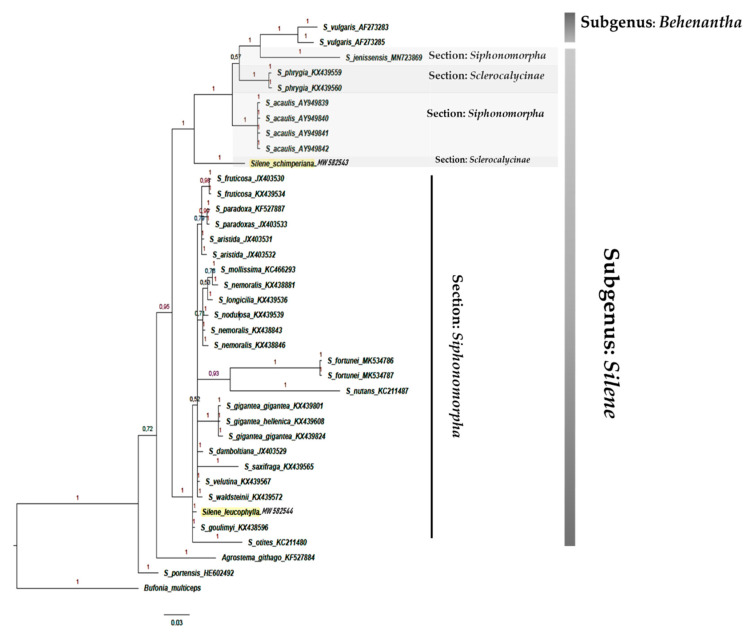
Bayesian phylogenetic tree of *Silene* species based on cpDNA sequences of the *psb*-A/*trn*-H marker. Numbers above branches represented (PP).

The phylogenetic tree based on the DNA sequences of the *psb*-A/*trn*-H showed that section *Sclerocalycinae* is not monophyletic. *Silene schimperiana* represented a sister clade to other *Silene* taxa belonging to section *Siphonomorpha* Otth: *S. acaulis* and *S. jenissensis*, and *S. vulgaris* related to the subgenus *Behenanthae*. Similarly, section *Siphonomorpha* was polyphyletic composed of 30 taxa, including the Egyptian species *S. leucophylla* (Figure 10).

**Figure 11:**
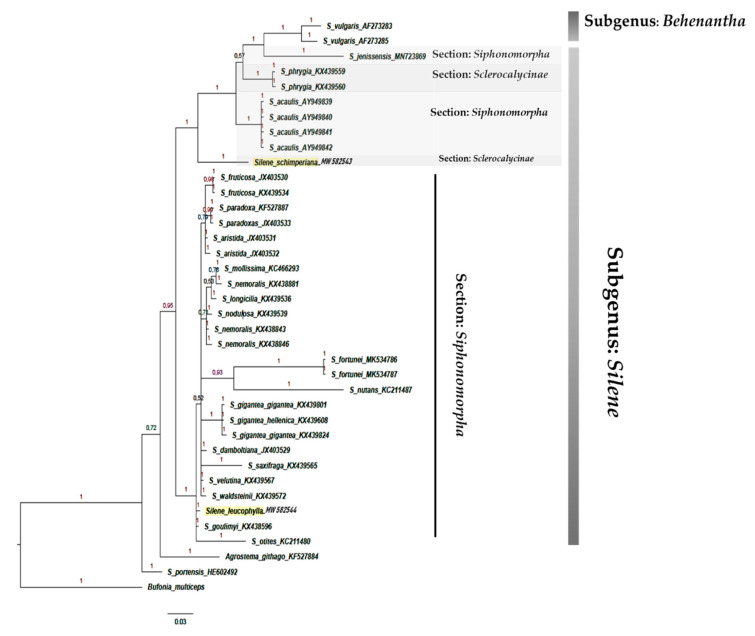
Bayesian phylogenetic tree of *Silene* species based on cpDNA sequences of the *rbc*L marker. Numbers above branches represented (PP).

The phylogenetic tree of the *rbc*L section *Sclerocalycinae* including *S. schimperiana,* represented a robust but unresolved clade (PP = 0.99). Simultaneously, Section *Siphonomorpha* including *S. leucophylla* was retrieved with a moderately supporting value PP = 0.88 (Figure 11).

**Figure 12:**
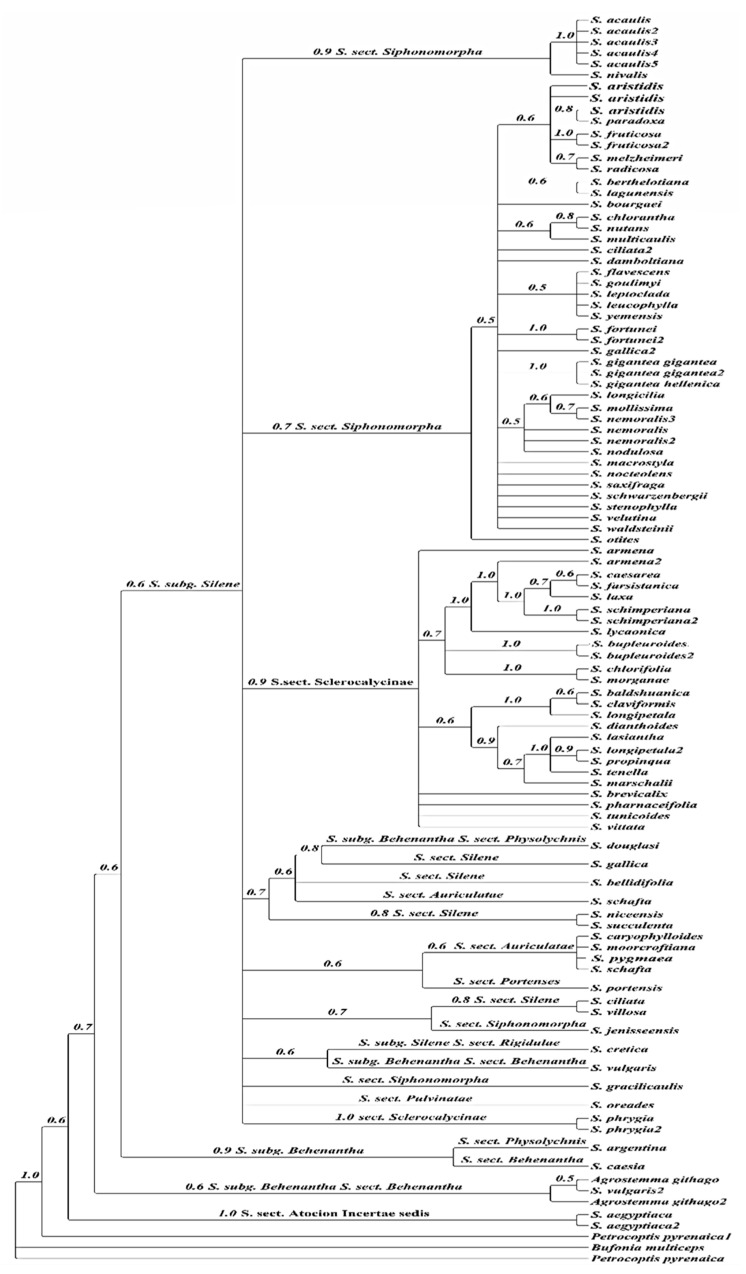
Bayesian cladogram of *Silene* taxa based on combined DNA sequences of ITS, *mat*K, *psb*-A/*trn*-H, and *rbc*L markers. Numbers above branches represented (PP).

In the four markers’ combined phylogenetic tree, the ingroup consisted of 102 taxa with 97 *Silene* taxa and five taxa from other genera (Figure 12). While *Bufonia multiceps* and *Petrocoptis pyrenaica* constituted the outgroup clades, *Agrostemma githago* was imbedded in the ingroup taxa.

The 97 ingroups of *Silene* taxa were divided into two subgenera: *Silene* subg. *Silene* Rohrbach, and *Silene* subg. *Behenantha* (Otth) Torr. & A. Gray, and neither of these two subgenera was monophyletic in our reconstruction. The subgenus *Silene* was composed of six sections: S*clerocalycinae, Siphonomorpha, Pulvinatae, Portenses, Auriculatae,* and *Atocion*, all of which were not monophyletic. *Silene schimperiana* and *S. leucophylla* clustered into their related sections as proposed by single markers analyses, Sect. *Sclerocalycinae* and Sect. *Siphonomorpha*, respectively.

## 3. Discussion

The current research represents the first detailed study of the micromorphological and molecular phylogenetic placement of the endemic *S. leucophylla* and the near-endemic *S. schimperiana* native to the Sinai Peninsula, Egypt.

Leaf tomentum and the stem wax secretion of *Silene* species have significant ecological and systematic importance concerning the interaction between plants and their environment [29]. The current study confirms the hairy leaf texture of *S. leucophylla*, whereas *S. schimperiana* was glabrous. In contrast, irregular epicuticular crustose wax platelets were observed on the stem and leaf of *S. schimperiana* only. The obtained results were consistent with [23], who indicated that where species grow in drier habitats, they can attain straight to curved anticlinal walls. *Silene leucophylla* and *S. schimperiana* have amphistomatic leaves. This is also a characteristic feature of species occupying xerophytic habitats [30].

Guard cells integrate multiple environmental signals and control the aperture width to ensure appropriate stomatal function for plant survival [31]. Therefore, guard cells have been studied extensively as a model system for scrutinizing environment sensing dynamics and mechanisms [25]. In *S. leucophylla*, the stem stomatal complex area measured 1.2 times larger than *S. schimperiana*. Quantitative characteristics of the leaf stomatal complex and the epidermal cells on the leaf’s AD were more extensive than those on the A.B. in both species. These indicate great integrity between the cell geometry and size of the epidermal cells and genetic constraints, and environmental factors [32,33].

The cell dimensions of the stomatal complex of *S. schimperiana* were 2.6–2.9 times larger than those of *S. leucophylla*. The stomatal pore and its area were 24.4–33.5 times larger *S. schimperiana* than in *S. leucophylla*. Additionally, the subsidiary cell area was 2.69–3.13 times larger in *S. schimperiana* than *S. leucophylla*.

As suggested by [34,35,36], plant species located in high-elevation and shady areas similar to the growing habitats of both *S. leucophylla* and *S. schimperiana* in the mountains of the Sinai Peninsula are usually characterized by a low average Stomatal Index (SI%). This critical trait (SI%) was 12.42% in *S. leucophylla* and 14.44% in *S. schimperiana*. The SI% for both species were considerably low compared to the high SI% (≥95.58%) reported in plant species growing in low-elevation and sunny habitats [22].

Rohrbach [6] classified *S. leucophylla* and *S. schimperiana* in the same section, “Botryosilene”. While he considered *S. leucophylla* in the Nutantes series, *S. schimperiana* was classified by him into the series *Sclerocalycinae*. Classification of the genus *Silene* by [10,11] considered *S. leucophylla* a member of section *Siphonomorpha*, while *S. schimperiana* was allied to section *Sclerocalycinae* (Subsection *Chlorifoliae*).

The *Sileneae* taxonomy and systematics were recently studied by [4,9,37], who integrated morphological and molecular phylogeny to reveal the phylogenetic relationships within the tribe *Sileneae*. According to [4,9], *S. leucophylla* belongs to the subsection *Brachypodae* (Boiss.) Gürke allied to section *Siphonomorpha*, while *S. schimperiana* belongs to subsection *Sclerocalycinae*.

In the current study, the phylogenetic analysis of each single and the four combined markers confirmed the generic, sectional and phylogenetic placement of the Egyptian species under investigation—with both *S. leucophylla* and *S. schimperiana* related to the subgenus *Silene*. At the same time, the sectional placement shows that *S. leucophylla* can be placed into the *S.* sect. *Sclerocalycinae* and *S. leucophylla* into *S.* sect. *Siphonomorpha*.

The phylogenetic trees’ topologies were generally consistent with the generic and sections of the *Silene* classification [4]. Most *Silene* sections were not monophyletic and the support values for clades were either weak or moderate. The clade support shows low support of section *Siphonomorpha* in the ITS analysis PP = 0.72 (Figure 8). In contrast, it was moderately supported in the *rbc*L analysis PP = 0.88 (Figure 1), and strongly supported (PP = 1) in both *mat*K (Figure 9) and *psb-*A/*trn-*H (Figure 10).

The results of Bayesian analysis of the ITS, *mat*K, and *rbc*L data sets supported the monophyly of the sections *Siphonomorpha* and *Sclerocalycinae*. However, *psb-*A/*trn-*H and the combined analysis failed to retrieve the monophyly of those sections.

According to [37], *S*. sect. *Siphonomorpha s.str*. was monophyletic and should be considered a separate section. The current study evaluated that this section should incorporates two complexes: the *Leucophylla* complex and the *Schimperiana* complex (Figure 8 and Figure 9).

The *Leucophylla* complex (Figure 8) was considered to be allied to the widely distributed Nutans group [38]. However, the current study reveals that the *Leucophylla* complex is confined to narrowly distributed taxa native to the South Mediterranean region, *S. flavescens*: Bulgaria, Greece, Hungary, Romania, and Yugoslavia; *S. leptoclada*: East Aegean Island, Turkey, and North Africa; *S. leucophylla*: endemic to Egypt; *S. yemensis*: Eritrea, Ethiopia, Saudi Arabia, and Yemen. Moreover, *S. saxifraga*, a native and widely distributed species in Europe, constitutes a sister group of the *Leucophylla* complex. In addition to the geographical distribution pattern, the current study confirms that members of the *Leucophylla* complex shared several morphological traits. Those traits were perennial life form, plant texture, stem woody base, leaf type, flowers number and inflorescence type, calyx shape, and capsule features [39,40,41,42].

Similarly, section Sclerocalycinae s.l. (Figure 8, Figure 9 and Figure 12) is composed of nine geographically restricted species of Silene: S. armena, S. caesarea, S. farsistanica, S. laxa, S. schimperiana, S. lycaonica, S. bupleuroides, S. chlorofolia and S. morganae. Meanwhile, the matK shows that this section is composed of four species: S. vittata, S. armena, S. schimperiana and S. tunicoides. These constitute a subset of Silene taxa native to the west Irano-Turinain floristic region, including Turkey, Armenia, Iran, and Iraq. These species establish the strongly supported Schimperiana complex (PP = 0.99, ITS; PP = 1, matK; and PP = 1 in the combined analysis). Members of the Schimperiana complex shared observable morphological traits: perennial life form, glabrous stem, leaf type “lanceolate to linear”, opposite cauline leaf arrangement, paniculate inflorescence, glabrous calyx, whitish-yellow and bifid petals, anthophore length from 1 mm to 15 mm, and capsule length from 3 mm to 15 mm [40,41,42,43].

## 4. Materials and Methods

### 4.1. Plant Materials

Two herbarium specimens of *Silene leucophylla* and three of *Silene schimperiana* were obtained from Assuit University Herbarium (ASTU). Three stem and leaf replicates of each specimen were used in SEM. For molecular analysis, fresh leaf materials were collected from two different localities at Saint Katherine, South Sinai, Egypt. Vouchers of the collected specimens are deposited at the herbarium of Suez Canal University, Ismailia, Egypt (SCUI) under the collection number (SCUI).

### 4.2. Specimens Examined

The following specimens were examined: *Silene leucophylla,* Egypt, Southern Sinai, Wadi Gebal, 28.3219 N, 33.5513 E, Alt. 1895–1991 m, 13 May 2004, Fayed, I. El-Garf, Abdel-Khalik and A. Osman; (ASTU#1); Egypt, Southern Sinai, Wadi Gebal, 28.3219 N, 33.5513 E, Alt. 1895–1991 m, 13 May 2004, Fayed, I. El-Garf, Abdel-Khalik and A. Osman; (ASTU#2); Egypt, Southern Sinai, Wadi Gebal, Reheibet Nada, 28.528761 N, 33.91720 E, Alt. 2099 m, 4 May 2016, Ahmed El-Banhawy, Ahmed Elkordy (SCUI-AEB#302); *Silene schimperiana*, Egypt, Southern Sinai Al-Meserdy, 28.3242 N, 33.5623 E, Alt. 1775–1940 m, 13 May 2004, A. Fayed, I. El-Garf, Abdel-Khalik and A. Osman, (ASTU#3); Egypt, Southern Sinai Al-Meserdy, 28.3242 N, 33.5623 E, Alt. 1775–1940 m, 13 May 2004, A. Fayed, I. El-Garf, Abdel-Khalik and A. Osman, (ASTU#4) Wadi Gebal, Farsh EL Rommanah, 28.536667 N, 33.901111 E, Alt. 2004 m, 4 May 2016, Ahmed El-Banhawy, Ahmed Elkordy, (SCUI-AEB#303); Southern Sinai Wadi Gebal, 28.3219 N, 33.5513 E, Alt. 1895–1919 m, 13 May 2016, A. Fayed, I. El-Garf, Abdel-Khalik and A. Osman, (ASTU#5).

### 4.3. Scanning Electron Microscopy (SEM)

The stem, as well as the leaf abaxial (AB) and adaxial (AD) surfaces, were mounted onto stubs with double-sided adhesive tape, coated for 5 min with gold in a polaron JFC-1100E coating unit, and then were examined and photographed with JEOL JSM-IT200 scanning electron microscope unit at Faculty of Science, Alexandria University, Alexandria, Egypt. The epidermal cell characteristics separately described for abaxial (AB) and adaxial (AD) leaf surfaces. The quantitative data of stomatal measurements were recorded in both closed and opened stomata, i.e., length, width, and area. The quantitative characteristics measured by image analysis software [44] followed the terminology in [29].

### 4.4. Statistical Analysis

Sixty-eight traits of the stem and leaf of the examined taxa were analyzed by using the R- software (Vienna, Austria), with the required packages installed [45]. Initially, boxplots were generated using the “*ggplot2*” library [46], to address the variations in the measured traits of the stem, and the AB and AD of the leaf. Analysis of variance (ANOVA) was performed using the (aov) function. Subsequently, post hoc Tukey Honestly Significant Difference (HSD) was used to figure out which group(s) of the sample differed [47]. The “*pheatmap*” and “*ggplot2*” packages [46,48] were used to visualize the similarity and dissimilarity within and among species. The scale of color is relative to the value of the divergence between investigated readings. The red color indicates the high similarity between accessions while the blue color assuming the low similarity [49]. The relationships among the quantitative traits were assessed through correlation analysis which visualized by correlogram using “corrplot2”. In the correlogram, the intensity of the colors is an indication for positive correlation, whereas the white box point indicates the insignificant correlation between variables [50].

### 4.5. DNA Extraction, PCR Amplification, Sequencing, and Phylogenetic Analysis

Two fresh leaf materials preserved in silica gel of each species were used for molecular analysis. Total DNA was extracted from silica-gel dried leaves using a silica column method similar to commercially available extraction kits [51]. DNA was extracted using the Cetyltrimethylammonium bromide (CTAB) protocol with some modifications [52]. The PCR amplification were performed in 15 µL volume for ITS, *mat*K, *rbc*L and *psb*-A/*trn*-H containing 5U/µL Taq DNA polymerase with 25 µM MgCl_2_, 10 µM of dNTPs, 10 µM of each primer. Amplifications were conducted using an Applied Biosystems^®^-VeritiTM 96- well thermal cycler (Thermo Fisher Scientific-Fisher Scientific AS-Postboks 114, Smestad-0309 Oslo, Norway). PCR products were purified with ExoSAP-IT (USB Corporation, Cleveland, OH, USA). PCR products were sent to Macrogen Spain for direct sequencing in both directions with an ABI 3730XL Genetic Analyzer (Life Technologies Corporation 5791 Van Allen Way Carlsbad, CA 92008). For the newly generated sequences, forward and reverse reads were assembled, and the contigs were edited into in GENEIOUS^®^ v.R9 (Biomatters Ltd., Berkeley, CA 94709-1405 USA https://www.geneious.com accessed 15 January 2021) using a personal license (C.A.). Four data matrices were constructed: ITS, *mat*K, *rbc*L, and *psb*-A/*trn*-H. The ingroup were selected to cover most of the major sections in the genus *Silene*. Each marker’s datasets were initially aligned using MAFFT algorithms, implemented in Geneious, using default alignment parameters, and visually revised to manually correct errors in alignment.

Eight novel DNA sequences of both species under investigation were deposited in the GenBank along with the following accessions: *S. leucophylla* (SCUI-AEB #302), ITS (MW 559753), *mat*K (MW 582539), *psb*-A/*trn*-H (MW 582543), and *rbc*L (MW 582541); and *S. schimperiana* (SCUI-AEB #303) ITS (MW 559754), *mat*K (MW 582540), *psb*-A/*trn*-H (MW 582544), and *rbc*L (MW 582542). Previously, published DNA sequences for ITS, *mat*K, *psb*A-*trn*H, and *rbc*L were downloaded from GenBank to construct balanced datasets, and NCBI codes are reported in the Supplementary (Table S3).

The optimal nucleotide substitution model was estimated using MrModeltest [53] and executed in MrBayes blocks. A 50% majority role consensus tree was constructed to get the posterior probabilities (PP). Posteriori probabilities, values >0.5 at a given branch were considered to support the existence of that branch [54]. All phylogenetic analyses were run on the CIPRES portal [55].

## 5. Conclusions

The combined investigations of the morphological data of leaf, stem and the molecular phylogenetic analysis of four molecular markers represented a comprehensive characterization of the endemic *S. leucophylla* and the near-endemic *S. schimperiana* native to Egypt, Yemen, and Saudi Arabia. The qualitative and quantitative morphological characters include presence/absence of trichomes, epicuticular wax type, leaf epidermal cells, and complex stomatal traits attributed entirely to well-characterizing endemic endangered species supported the previous taxonomic revisions. The Bayesian phylogenetic inference of *S. leucophylla* and *S. schimperiana* using four nuclear markers portrayed the target taxa’s phylogenetic position within the whole genus. Both species related to the subgenus *Silene* and their corresponding sections, where *S. leucophylla* allied to section *Siphonomorpha* and *S. schimperiana* allied to section *Sclerocalycinae*.

The sectional classification of the *Silene* species native to the Sinai Peninsula, Egypt has so far always been investigated on the basis of morphological descriptions only. The current research represents the first phylogenetically based study to reveal the sectional classification of *S. leucophylla* and *S. schimperiana*. Moreover, two morphologically and phylogenetically supported *Silene* complexes are proposed by the current research. The *Leucophylla* complex allied to section *Siphonomorpha* and the *Schimperiana* complex related to section *Sclerocalycinae*. However, these two complexes require further investigation and more exhaustive sampling to infer their complex phylogenetic relationships.

## Figures and Tables

**Table 1:** Qualitative characteristics of stem and leaf Abaxial leaf (AB) and Adaxial leaf (AD) characters of *Silene leucophylla* and *Silene schimperiana*.

Characters	*Silene leucophylla* Boiss.	*Silene schimperiana* Boiss.
**Stem characters**		
Surface	Pubescent	Glabrous
Epicuticular Wax	Thin layer	Irregular crustose platelets
Trichomes	Present	Absent
Trichomes Type	Unicellular, non-glandular	Absent
Trichome Surface	Densely pustulate	Absent
Trichomes Length	45–98 µm	Absent
Trichomes Width	13–23 µm	Absent
Stomata Type	Anomocytic	Anomocytic
**Abaxial leaf (AB) characters**		
Epidermal Cell Shape	Irregular oblong to bone-shape	Parallel tetra- to polygonal
Anticlinal Walls	Sunken channeled irregularly curved	Sunken straight
The curvature of Outer Periclinal Walls	Convex	Slightly Convex
Fine Relief of the Cell Wall	Highly ribbed	Irregular epicuticular crustose platelets
Trichomes Type	Unicellular, non-glandular	Absent
Trichome Surface	Densely irregular pustulate	Absent
Trichomes Length	78–130 µm	Absent
Trichomes Width	18–20 µm	Absent
Stomata Type	Diacytic	Diacytic
Stomata Level	Raised	Sunken
Guard Cell Surface	Smooth	Irregular epicuticular crustose platelets
Pore Shape	Elliptic slit	Linear slit
**Adaxial leaf (AD) characters**		
Epidermal Cell Shape	Irregular oblong to bone-shape	Parallel penta- to polygonal
Anticlinal Walls	Sunken channeled irregularly curved	Sunken straight
The curvature of Outer Periclinal Walls	Convex	Slightly Convex
Fine Relief of the Cell Wall	Moderately ribbed	Irregular epicuticular crustose platelets
Trichomes Type	Unicellular, non-glandular	Absent
Trichome Surface	Densely irregular pustulate	Absent
Trichomes Length	75–125 µm	Absent
Trichomes Width	16–25 µm	Absent
Stomata Type	Diacytic	Diacytic
Stomata Level	Raised	Sunken
Guard Cell Surface	Smooth	Irregular epicuticular crustose platelets
Pore Shape	Elliptic slit	Elliptic + Linear slits

## Data Availability

The data presented in this study are available in this article and Supplementary Material.

## Supplementary Materials

The following are available online at https://www.mdpi.com/article/10.3390/plants10040740/s1. Table S1. Morphological differences between *Silene leucophylla* and *Silene schimperiana* and the nearest elements of the genus *Silene*. Table S2. Quantitative characteristics of stem and leaf Abaxial leaf (AB) and Adaxial leaf (AD) characters of *Silene leucophylla* and *Silene schimperiana*. Table S3. SEQs Accession numbers of ITS, *mat*K, *psb*A-*trn*H, and *rbc*L downloaded from GenBank.
