# Supplementary material for: Taxonomic Revisiting and Phylogenetic Placement of Two Endangered Plant Species: Silene leucophylla Boiss. and Silene schimperiana Boiss. (Caryophyllaceae)
Source: Plants (Basel). 2021 Apr 9;10(4):740. doi: 10.3390/plants10040740 (PMC8070032; doi:10.3390/plants10040740)
Supplement: Supplementary file 1 [file plants-10-00740-s001.pdf]

Table S1. Morphological differences between *Silene leucophylla* and *Silene schimperiana* and the nearest elements of the genus *Silene*.

Species Name	Stem	Leaves	Inflorescence	Flowers	Capsule
Silene leucophylla	Viscid, Perennial, tomentose-canescent with woody structure at the base up to 30 cm long. Stem, erect, hairy, cylindrical, internode length 2–5 cm, stems to 40 cm.	Basal leaves, pubescent densely rosette ovate to spathulate, rarely orbicular 0.6–3 × 0.1–0.8 cm; petiole 0.4–3 cm. long, olive color, with acute apex and entire margins. Cauline leaves ovate to spathulate 0.3–1.2 × 0.1–0.5 cm. opposite decussate, petiolate, with acute apex and entire margins.	Inflorescence cymose; with 3-5 flowers, pedicelled; Pedicel length 0.4–0.7 cm.	Pedicel to 7mm; calyx oblong-cylindrical, 1.2-1.9 x 0.1-0.3cm., 10 nerved, hairy, calyx teeth triangular or ovate-deltoid, obtuse, with scarious margins; ca. 3 mm long. Petals 5 purple to whitish violet to violet, 2.8–5.5 cm long (with claw length 0.5–0.7 cm and limb 2–2.3 cm. long). Carpophore equaling the capsule.	Capsule oblong ovoid capsule 1.9 cm. long
Silene leptoclada	Caespitose perennial, stems erect, slender, 25-35 cm, with an indumentum of short, spreading hairs.	Basal leaves rosulate, oblanceolate-spathulate, canescent sericeous; cauline leaves narrowly oblanceolate to very narrowly elliptic, distant.	Inflorescence a dichasium, the main axis terminating in a flower. 2-5 flowers	Calyx 11.5-15 mm, with an indumentum of short glandular hairs, 10-nerved, the teeth broadly triangular. Petals bifid. petal limb yellowish Anthophore 2.5-3.5 mm.	Capsule oblong, 8-9.5 mm.

Silene flavescens	Caespitose perennial, stems erect, slender, 25-35 cm, with an indumentum of short, spreading hairs.	Basal leaves rosulate, oblanceolate-spathulate, canescent sericeous; cauline leaves narrowly oblanceolate to very narrowly elliptic, distant.	Inflorescence very reduced and raceme-like	Calyx 9-10 mm, very shortly puberulous, anthophore ca. 2 mm. Petal yellowish white to greenish- yellow above	Capsule oblong, 8-9.5 mm.
Silene yemensis	Densely villous perennial; stems 10-45cm, arising from a woody base, densely villous below, glandular-villous above.	Basal leaves arranged in rosettes, leaves pubescent; the lamina broadly cuneate, 15-30 x 10-20mm, apiculate, villous, more densely so on the margins and midrib, glandular above, contracted below into a 10-40mm petiole; cauline leaves much reduced sessile, linear-lanceolate.	Flower solitary or in 2-5-flowered lax dichasial cymes; pedicels up to 20mm, glandular-villous.	Calyx 10-ribbed, 13-15mm, white, gland, slightly inflated in fruit, glandular-villous, especially on the ribs; calyx teeth triangular-lanceolate, ca. 2mm, acute. Corolla pink to brownish, c.20mm; limb ca. 7mm, glabrous, divided in the top third into 2 linear lobes. carpophore 4-6mm, glabrous.	Capsule ovate ll-12mm long;

Silene saxifraga	Perennial, pubescent at base, carpet-forming, woody rootstock; 10-20 cm, many stems, spindly, viscous at the top; caudiculis suffruticosae complicated prostrales, stems are slender filiform	Leaves linear or linear-lanceolate, pointed ciliated, rough edges	Two or dichotomous, solitary or twinned, 2-3 flower peduncle long	Flowers greenish-white above, reddish-green underneath, calyx club-shaped, vestige of calyx, glabrous, whitish, 10 branching veins, oval-obtuse teeth; bifid petals, crowns of obtuse scales, no auricle to body, but ciliated; the delicate glabrous carpophore equaling capsule. anthophore 3-4 mm	Capsule ovoid or oblong,
Silene oreosinaica	Perennial, with woody structure at the base up to 25 cm. long. Stem, hairy, cylindrical; internode up to 5 cm long.	Basal leaves lanceolate, 0.4-4.5 x 0.1-0.9 cm. arranged in rosette shape with acute apex, entire margins, symmetrical base and sessile. Cauline leaves lanceolate, smaller than basal leaves 0.4-2.5 x 0.1-0.2 cm, opposite decussate with acute apex, entire margin, symmetrical base and sessile	Inflorescence racemes, pedicle; Pedicel length 0.5 cm long.	Calyx tubular 0.8 -1.2 cm long, 10 nerved with yellow nerve, hairy. Bract lanceolate. Petals 5 white in color with very pale pink edges 1.1-1.9 cm long (claw 0.4-0.6 cm long and limp 0.7-1.2 cm long). Petals that exposed to the sun are converted to rose color	Capsule ovoid 0.8 -1.2 cm. long.

Silene schimperiana	Perennial glaucous, herb, woody at the base, stems 50-80cm, erect rigid branching thicker at the nodes	Basal leaves 4-10 x 0.3-0.5cm, narrowly linear-spathulate, acute, with a prominent midrib on the lower surface. Cauline leaves linear to spathulate, opposite decussate, with acute apex, entire margin, symmetrical base.	Flowers in lax paniculate cymes, solitary or rarely 2 together, subtended by 2-minute bracteoles; pedicel 1.5-2cm;	Calyx oblong-cylindrical 2-2.5 mm, 10 nerved, glabrous, dimorphic, calyx teeth, triangular with acute apex 2–3 mm. long; Petals 2-fids, Petals five white in the upper side and yellowish in the back side, sometimes with violet nerves, 3.5-5.2 cm. long (with claw length 1.5 - 2.7 cm and limp 2 -2.7 cm.); carpophore equaling the capsule.	Capsule 1.3-1.5cm, oblong, glabrous
Silene armena	Caespitose perennial, stems up to 50 cm, stems glabrous, viscid	Basal leaves linear to oblanceolate, narrow. Cauline leaves linear, very narrow, margins serrulate or papillose-scabrid above.	Inflorescence racemose, sometimes paniculately branched. Bracts linear.	Calyx 13-18(20) mm. Petals bipartite to the middle, whitish or yellowish. glabrous calyx. Anthophore (4)5-9 mm.	Capsule 9-15 mm, sometimes slightly exerted
Silene tunicoides	Perennial with many erect, slender stems arising from a very woody branched base. Stems up to 30 cm, with short, retrorse indumentum below, glabrous, and viscid above.	Cauline leaves linear, setaceous, 10-15 mm, margins setose.	Inflorescence paniculate, the branches wide-spreading and cymose.	Calyx 3-4 mm, glabrous, ovoid. Petals yellowish green, with an entire limb. Anthophore 1 mm or less.	Capsule 3-4 mm, included in the calyx.

Silene vittata	Rigid annual. Stems erect, up to 50 cm, puberulent below, glabrous and often viscid above.	Basal leaves forming a rosette, spathulate, petiolate, often puberulent; cauline leaves linear to linear-lanceolate.	Inflorescence richly dichasial branched.	Calyx 11-15 mm, nerves conspicuous, the teeth broadly triangular with dense, anastomosing venation, glabrous. Petals pink, conspicuously purple-veined outside; coronal scales dentate-incised. Filaments glabrous. Anthophore 6-10 mm.	Capsule ovoid-globose, 7-8 mm.
Silene caesarea	Perennial, up to 80 cm, viscid above.	Basal leaves small, soon withering. Cauline leaves more than 3 x longer than broad, lanceolate to oblong-lanceolate, acute, cordate at the base, more than 5 mm broad.	Inflorescence a spreading panicle	Calyx 20-22 mm. Petal limb bipartite to the middle into oblong lobes. Anthophore 8-10 mm, shorter than the exserted capsule. Petals white	Capsule oblong, exerted from calyx

Silene farsistanica	Perennial. Up to a few more woody 100 cm tall,	pale green, sometimes purple-and rosettes of leaves suffused. Individual or more. Basal 32- 4- 75 × 10 mm, sub-spathulate as far as oblanceolate, attenuate into a petiole; cauline leaves (36) 30 - 56 (70) × 5 - 9 mm oblong-oval or oblong-lanceolate petiolate or nearly acute; All the leaves are fleshy.	Inflorescence panicle flowered spreading. Bracts 3-5 cm long, linear triangular, sharp, and the wider towards the base of the hyaline margined.	Calyx 17, 5-26,5mm long, cylindrical, green colored, often violescent. The teeth are alternately 2,5-4,5 mm long, obtuse and acute angle ± Peri-wide margins. Petals white or yellow; limb c. 10 mm long, fingernail c. 13 mm high.	Capsule 11 - 17 × 4,5 - 6 mm long, ovoid, slightly protruding from the calyx;
Silene laxa	Glaucous, glabrous perennial. Stems erect, viscid above, 70-100 cm.	Basal leaves small, soon withering. Median cauline leaves up to 2 (2.5) x longer than broad, ovate to elliptic, cordate at the base, acute, more than 10 mm broad.	Inflorescence racemose or paniculate, lax.	Calyx 12-23 mm. Petals white to khaki, the limb bipartite into oblong-obovate lobes. Anthophore 6-9.5 mm. glaucous calyx	Capsule 13-17 x 5-6 mm, somewhat exerted from the calyx
Silene lycaonica	Caespitose, glabrous perennial. Stems erect, c. 25 cm.	Leaves dimorphic, the basal obovate to broadly elliptic, fleshy, the cauline very reduced, linear to lanceolate.	Inflorescence racemose, few-flowered.	Calyx 12-16 mm. Petal limb divided to beyond the middle into oblong lobes. Anthophore 6-7 mm.	Capsule 7-8 x 4 mm, included in the calyx.

Table S2. Quantitative characteristics of stem and leaf Abaxial leaf (AB) and Adaxial leaf (AD) characters of *Silene leucophylla* and *Silene schimperiana*.

Character	Abbreviations	*Silene leucophylla*							*Silene schimperiana*						
		Mean	SD	Median	Min	Max	Range	SE	Mean	SD	Median	Min	Max	Range	SE
**Stem characters**															
**Stomatal Pore Length**	SPL	10.57	2.5	10.09	7.67	14.4	6.73	1.12	13.55	2.81	13.32	10.85	16.47	5.62	1.63
**Stomatal Pore Width**	SPW	1.89	0.87	1.33	1.16	2.88	1.71	0.39	1.47	0.46	1.72	0.95	1.75	0.8	0.26
**Stomatal Pore Area**	SPA	14.19	7.09	12.33	7.38	25.1	17.72	3.17	14.71	8.04	18.75	5.45	19.93	14.48	4.64
**Stomatal Complex Length**	SCL	21.9	2.16	21.9	20.37	23.43	3.05	1.53	19.05	2.67	17.78	17	23.49	6.49	0.94
**Stomatal Complex Width**	SCW	5.66	1.59	5.66	4.53	6.78	2.25	1.13	6.21	1.11	6.02	4.65	7.97	3.32	0.39
**Stomatal Complex Area**	SCA	107.40	29.41	107.4	86.61	128.19	41.59	20.79	88.84	17.83	89.65	63.05	119.72	56.66	6.31
**Abaxial leaf (AB) characters**															
**Stomatal Pore Length**	SPL.AB	9.34	1.36	8.89	8.41	11.74	3.33	0.61	15.33	1.25	15.67	13.6	17.15	3.55	0.47
**Stomatal Pore Width**	SPW.AB	1.82	0.5	1.84	1.03	2.33	1.3	0.22	1.72	0.66	1.49	1.06	2.62	1.56	0.25
**Stomatal Pore Area**	SPA.AB	11.79	5.11	13.92	5.36	17.38	12.02	2.29	15.26	5.66	13.76	9.36	23.84	14.48	2.14
**Stomatal Complex Length**	SCL.AB	15.18	2.61	14.28	11.84	18.19	6.35	0.87	22.14	1.8	22.56	19.19	23.69	4.5	0.81
**Stomatal Complex Width**	SCW.AB	5.37	0.6	5.6	4.51	6.13	1.63	0.2	8.55	1.25	8.31	7.06	10.51	3.45	0.56
**Stomatal Complex Area**	SCA.AB	61.42	8.56	63.79	46.44	74.64	28.21	2.85	151.82	21.4	149.01	125.33	185.13	59.8	9.57
**Number of Lobes**	NLP.AB	8.33	1.66	9	6	11	5	0.55	6.5	1.07	6	5	8	3	0.38
**Epidermal Cell Length**	ECL.AB	34.74	7.71	35.96	23.51	46.04	22.53	2.57	47.57	11.36	47.18	30.52	61.4	30.88	4.01
**Epidermal Cell Width**	ECW.AB	16.09	3.97	16.87	8.73	21.95	13.22	1.32	52.39	10.4	53.69	37.85	63.79	25.94	3.68
**Subsidiary Cell Length**	SBL.AB	41.43	4.02	42.44	32.66	45.72	13.06	1.34	56.58	5.03	56.89	50.77	61.77	11	2.52
**Subsidiary Cell Width**	SBW.AB	24.25	4.24	23.31	18.13	30.58	12.45	1.41	45.01	8.12	45.35	36.43	52.92	16.49	4.06
**Subsidiary Cell Area**	SBA.AB	740.64	201.85	767.86	450.11	1049.28	599.17	67.28	2306.28	920.76	2328.15	1287.04	3281.8	1994.76	460.38
**Number of Stomata**	SCN.AB	10	2.83	10	8	12	4	2	5	1.41	5.5	3	6	3	0.71
**Number of Epidermal Cells**	NEC.AB	73.5	19.09	73.5	60	87	27	13.5	30.75	2.22	31	28	33	5	1.11
**Stomatal Index**	SI.AB	11.94	0.25	11.94	11.76	12.12	0.36	0.18	13.78	2.81	14.84	9.68	15.79	6.11	1.41
**Adaxial leaf (AD) characters**															
**Stomatal Pore length**	SPL.AD	9.95	0.68	10.11	8.99	10.58	1.59	0.34	13.8	3.26	12.73	8.94	18.45	9.52	1.03
**Stomatal Pore width**	SPW.AD	1.67	0.42	1.75	1.15	2.03	0.88	0.21	2.29	0.62	2.14	1.67	3.58	1.91	0.2
**Stomatal Pore area**	SPA.AD	13.49	2.55	14	10.13	15.84	5.7	1.27	21.42	8.55	20.4	12.26	36.7	24.44	2.7
**Stomatal Complex length**	SCL.AD	16.32	1.4	16.38	14.29	18.71	4.42	0.39	20	1.25	19.94	17.9	22.51	4.61	0.36

**Stomatal Complex width**	SCW.AD	5.35	0.77	5.19	3.6	6.77	3.17	0.21	9.53	1.98	9.37	6.19	13.33	7.14	0.57
**Stomatal Complex area**	SCA.AD	64.85	9.97	60.12	54.65	81.03	26.38	2.77	152.3	37.92	147.69	102.2	253.5	151.3	10.95
**Number of lobes**	NLP.AD	8.55	1.29	8	7	11	4	0.39	7.07	1.28	7	5	10	5	0.33
**Epidermal Cell length**	ECL.AD	43.2	5.94	44.19	31.16	52.41	21.25	1.79	57.61	15.76	57.53	35.48	91.04	55.56	4.07
**Epidermal Cell width**	ECW.AD	19.6	5.45	18.25	12.79	26.97	14.19	1.64	48.83	16.59	43.97	32.47	80.59	48.12	4.28
**Subsidiary Cell Length**	SBL.AD	47.76	5.25	47.76	38.05	58.57	20.52	1.52	76.92	18.43	71.6	54	117.97	63.97	4.61
**Subsidiary Cell Width**	SBW.AD	28.14	4.19	28.18	21.69	35.43	13.75	1.21	48.82	10.38	47.58	32.69	62.67	29.97	2.59
**Subsidiary Cell Area**	SBA.AD	1049.08	167.81	1074.53	740.86	1304.84	563.97	48.44	2863.33	663.9	2778.23	1782.32	4048.75	2266.43	165.98
**Number of Stomata**	SCN.AD	8	1	8	7	9	2	0.58	4.75	0.96	4.5	4	6	2	0.48
**Number of Epidermal Cells**	NEC.AD	54.67	5.69	53	50	61	11	3.28	27	3.27	27	23	31	8	1.63
**Stomatal Index**	SI.AD	12.89	2.49	13.11	10.29	15.25	4.96	1.44	15.1	3.76	13.4	12.9	20.69	7.79	1.88

Table S3. Sequences accession numbers of ITS, *mat*K, *psb*A-*trn*H, and *rbc*L downloaded from GenBank.

**Species**	GenBank accession numbers			
	**ITS**	***mat*k**	***rbcL***	***Psb*-A/*trn*-H**
Agrostema githago	-	AY936315	MK895562	KF527884
Bufonia multiceps	-	-	KX709610	MW805351
Petrocoptis pyrenaica	FJ384018	FJ589508	-	-
Petrocoptis viscosa	KF728864	-	-	-
Petrocoptis glaucifolia	KF274510	-	-	-
S. acaulis	KX757546	FJ404872	KC484097	AY949839
S. acaulis	-	-	KF602214	AY949840
S. acaulis	-	-	-	AY949841
S. acaulis	-	-	-	AY949842
S. aegyptiaca	KX757411	-	-	-
S. aegyptiaca	KX757417	-	-	-
S. argentina	FJ589513			
S. aristidis	KX757466	-	-	JX403531
S. aristidis	-	-	-	JX403532
S. armena	KX757619	FJ589514	-	-
S. baldshuanica	KX757615	-	-	-
S. bellidifolia	-	FJ589516	-	-
S. berthelotiana	-	MN783968	MN783857	-
S. bourgaei	-	MN783969	-	-
S. brevicalix	KX757624	-	-	-
S. buplerioides	MN420822	-	-	-
S. bupleuroides	LC424085	-	-	-
S. caesarea	MK530524	-	-	-
S. caesia	-	FJ589517	-	-
S. caryophylloides	-	FJ589518	-	-
S. chlorantha	KX757527	-	-	-
S. chlorifolia	MK554637	-	-	-
S. ciliata	KP849853	-	KM200337	-
S. ciliata	-	-	KM200343	-
S. ciliata	-	-	-	-
S. claviformi	KX757632	-	-	-
S. cretica	-	-	EF418560	-
S. damboltiana	-	-	-	JX403529
S. dianthoides	KX757626	-	-	-
S. douglasii	-	-	MG249241	-
S. farsistanica	MK554636	-	-	-
S. flavescens	KP849847	FJ589526	-	-
S. fortunei	MH808304	MK534845	MK534836	MK534786
S. fortunei	-	-	-	MK534787
S. fruticosa	KX757507	FJ589527	-	JX403530

S. fruticosa	-	-	-	KX439534
S. gallica	-	FJ589528	MF065146	-
S. gallica	-	-	MH658273	-
S. gigantea ssp. gigantea	-	-	-	KX439801
S. gigantea ssp. gigantea	-	-	-	KX439824
S. gigantea ssp. hellenica	-	-	-	KX439608
S. goulimyi	-	-	-	KX438596
S. gracilicaulis	-	FJ589530	-	-
S. jenisseensis	-	MH714125	-	-
S. jenisseensis	-		MH713910	-
S. jenissensis	-	MN723869	-	-
S. lagunensis	-	MN783971	-	-
S. lagunensis	-	-	KJ595785	-
S. lasiantha	MN447216	-	-	-
S. laxa	MK554642	-	-	-
S. leptoclada	KX757520	-	-	-
S. leucophylla	MW 559753	MW582539	MW582541	MW582543
S. longicilia	-	-	-	KX439536
S. longipetala	KX757616	-	-	-
S. longipetala	MK542829	-	-	-
S. lycaonica	KX757621	-	-	-
S. macrostyla	KX757535	-	-	-
S. marschalii	MK559500	-	-	-
S. melzheimeri	KF267889	-	-	-
S. mollissima	-	-	-	KC466293
S. moorcroftiana	-	FJ589541	-	-
S. morganae	MK530513	-	-	-
S. multicaulis	KX757469	FJ589542	-	-
S. nemoralis	-	-	-	KX438843
S. nemoralis	-	-	-	KX438846
S. nemoralis	-	-	-	KX438881
S. niceensis	-	FJ589545	-	-
S. nivalis	KX757557	-	-	-
S. nocteolens	-	MN783972	-	-
S. nodulosa	-	-	-	KX439539
S. nutans	KX757472	-	-	KC211487
S. oreades	KX757579	-	-	-
S. otites	MT796574	AY514848	KC171366	KC211480
S. otites	-	FJ589547	KJ746297	-
S. otites	-	-	KF997306	-
S. paradoxa	JX403524	KF527887	KF527887	KF527887
S. paradoxa	-	FJ589548	-	JX403533
S. pharnaceifolia	KX757631	-	-	-

S. phrygia	-	-	-	KX439559
S. phrygia	-	-	-	KX439560
S. pygmaea	-	FJ589551	-	-
S. portensis	EF060237	-	-	HE602492
S. portensis	-	-	-	-
S. propinqua	MK554639	-	-	-
S. radicosa	KX757470	-	-	-
S. saxifraga	KX757493	KC171400	-	KX439565
S. schafta	AJ831792	FJ589556	EF418563	-
S. schimperiana	MW 559754	MW582540	MW582542	MW582544
S. schimperiana	-	-	MF668591	-
S. schwarzenbergii	-	FN821199	-	-
S. schwarzenbergii	-	FJ589557	-	-
S. stenophylla	KX757536	-	-	-
S. succulenta	-	FJ589562	-	-
S. tenella	MK559502	-	-	-
S. tunicoides	KX757629	FJ589563	-	-
S. velutina	-	-	-	KX439567
S. villosa	-	-	EF418563	-
S. vittata	-	FJ589566	-	-
S. vulgaris	AY594309	-	EU677008	AF273283
S. vulgaris	-	-	HE574592	AF273285
S. waldsteinii	KX757471	-	-	KX439572
S. yemensis	KX757471	FJ589566	-	-
